# Genome-Scale Phylogenetic Analyses Provide Insights into the Phylogenetic Placement of *Fusarium commune*

**DOI:** 10.3390/jof12020112

**Published:** 2026-02-05

**Authors:** Shunsuke Nozawa, Yosuke Seto, Kyoko Watanabe

**Affiliations:** 1 School of Agriculture, Tamagawa University, Machida, Tokyo 194-8610, Japan; 2 Cancer Chemotherapy Center, Japanese Foundation for Cancer Research, 3-8-31, Ariake, Koto-Ku, Tokyo 135-8550, Japan

**Keywords:** *Fusarium*, species complex, evolutionary history, concatenation approach, coalescence approach, monophyly

## Abstract

Recent advances in high-throughput sequencing technologies have significantly enhanced the accuracy of phylogenetic inference, enabling comprehensive genome-wide analyses. *Fusarium* fungi, which include numerous agriculturally and medically important species, are typically classified at the species complex (SC) level. Clarifying the evolutionary relationships and distinctiveness of these SCs is therefore essential for accurate identification and understanding of their biology. Recent large-scale phylogenetic studies based on genomic data have provided a more resolved understanding of the evolutionary relationships among *Fusarium* SCs, supporting the view that most represent evolutionarily coherent and stable lineages. However, the phylogenetic position of *Fusarium commune* has not been explicitly examined, despite incongruence between phylogenies inferred from nucleotide and amino acid sequence data. This study aimed to clarify the phylogenetic placement of *F. commune* at the SC level by re-examining its position using a genome dataset independent of those employed in previous studies. Our results are largely consistent with previously reported genome-scale phylogenetic analyses of the genus *Fusarium* and support the stability of most SCs. However, *F. commune* was not clearly included in any of the currently recognized SCs and instead formed an independent lineage. These findings provide insights into the evolutionary history of *Fusarium* SCs and contribute to a better understanding of the taxonomic position of *F. commune*.

## 1. Introduction

Recent developments in next-generation sequencing technologies, methodologies, and software have enabled the analysis of genome-wide phylogenies. Genome-scale phylogenetic trees constructed from the alignment of hundreds to thousands of concatenated genes can significantly improve the reliability of phylogenetic tree branches and provide valuable insights into phylogenetic relationships [1]. Additionally, software for genome-scale coalescent-based phylogenetic analyses, such as ASTRAL [2], has been developed and used to resolve controversial lineages [3]. Furthermore, genome data allows us to predict polytomies, which are useful for discussing evolutionary histories [4,5].

The taxonomy of *Fusarium* Link has long been studied by many researchers because *Fusarium* fungi are internationally recognized as important phytopathogenic and clinical fungi. The genus *Fusarium* was established in 1809, and since nucleotide-sequence-based phylogenetic analyses became possible, approximately 400 species are now recognized phylogenetically.

In recent years, taxonomic studies of *Fusarium* have focused on ongoing debates regarding the circumscription of the genus. Earlier phylogenetic analyses based on multiple loci were often limited by low branch support, which hindered the clear delimitation of generic boundaries [6,7,8,9]. Recent genome-scale phylogenetic analyses have substantially improved branch support for the *Fusarium* sensu stricto clade as well as the Fusarium terminal clade (FTC), thereby providing a robust framework for taxonomic inference [10,11]. Genome-scale phylogenetic approaches have also been applied to evaluate the monophyly of species complexes (SCs). Gómez-Chavarría et al. [10] demonstrated the monophyly of SCs using genome-wide datasets based on amino acid sequences. Similarly, Lizcano-Salas et al. [11], using nucleotide-based genomic data, supported these findings and further reassessed the branching order and monophyly of the SCs. Based on the analyses of the above genome-scale datasets, Ulaszewski et al. [12] demonstrated that genome size, lactic enzymes, biosynthetic gene clusters of secondary metabolites, and the repertoire of small secreted proteins reflect the evolutionary history of Fusarium sensu stricto and its related lineages positioned in ancestral clades.

**Figure 1:**
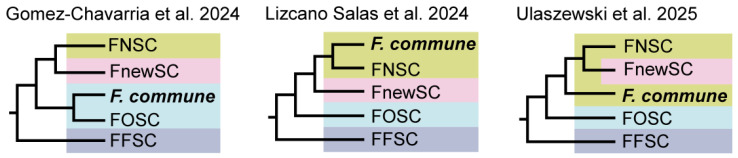
Comparison of placement of *F. commune* in genome-scale phylogenetic tree in previous studies ([10,11,12]). The bold text indicates the phylogenetic position of *F. commune*. The yellow background indicates the *Fusarium nisikadoi* species complex (FNSC), the pink background indicates *F. newnesense* SC (FnewSC), the blue background indicates *F. oxysporum* SC (FOSC), and the navy background indicates *F. fujikuroi* SC (FFSC).

However, the phylogenetic placement of *Fusarium commune* has remained inconsistent even in genome-scale phylogenetic analyses, depending on the data type (amino acid and nucleotide sequences) (Figure 1). Gómez-Chavarría et al. [10] placed *F. commune* within the *F. oxysporum* SC, as a lineage closely related to members of that complex based on amino acid data. In contrast, Lizcano-Salas et al. [11] classified *F. commune* within the *F. nisikadoi* SC, positioning it as a lineage closely related to *F. nisikadoi* SC based on nucleotide data. By using amino acid data, Ulaszewski et al. [12] further placed *F. commune* as a lineage adjacent to a clade comprising *F. nisikadoi* SC and the *F. newnesense* SC, treating it as paraphyletic with respect to that clade but nevertheless considering it part of *F. nisikadoi* SC.

These discrepancies have left the taxonomic placement of *F. commune* unresolved. Therefore, the present study aims to clarify the phylogenetic position of this species and to reassess the monophyly of the SCs in *Fusarium*. To this end, we conducted genome-scale phylogenetic analyses using both nucleotide and amino acid sequence data, applying concatenation-based and coalescent-based methods. Furthermore, based on the resulting phylogenies, we calculated concordance factors (gCF and sCF) and performed polytomy tests to discuss the phylogenetic support and branching patterns of the *F. commune* and its related SCs.

## 2. Materials and Methods

### 2.1. Taxon Sampling

The ingroup taxa comprised genomic data from 101 species (previously identified), representing 23 *Fusarium* SCs. As outgroup taxa, three strains belonging to three species of *Neonectria* (*N. coccinea*, *N. galligena*, and *Nectria* sp.) and *Trichoderma brevicompactum* were selected based on previous studies (Supplementary Table S1 [6,7,13,14,15,16,17,18,19,20,21,22,23,24,25,26,27,28,29,30]).

### 2.2. Orthology Inference

Gene predictions were performed using Augustus v.3.3.3 [31] with the parameter: “--genemodel = complete --species = fusarium <genomic data>”. OrthoFinder v2.5.4 [32] was used to identify single-copy orthologs shared among taxa using default parameters (inflation parameter 1.5). Obtained single-copy orthologs were aligned with MAFFT v7.505 [33] with default setting. Gaps including sites in alignments were removed using trimAl v1.415 [34] with the command option “-g 10”.

Genes longer than 1000 bp (excluding gap-containing sites after alignment) were selected, as Mirarab et al. [2] demonstrated that species tree accuracy improves when individual gene lengths increase from 500 to 1000 bp. Sequence statistics for the concatenated datasets were calculated using the Sequence Data Explorer module of MEGA10 [35].

### 2.3. Phylogenetic Analyses

Phylogenetic trees were constructed using concatenation and coalescence approaches. Concatenated alignments of DNA and amino acid data from all gene sets (868 genes) were analyzed using IQ-TREE v.2.0.7 [36] under the GTR + G + I and LG models, respectively, with 1000 bootstrap replicates.

For the coalescence approach, species trees were inferred using the summary-tree method implemented in ASTRAL III v.4.4.4 [2] with the parameter: “-i <gene trees data> -o <species tree> -t 3”. Individual gene trees were first constructed from the DNA and amino acid datasets using IQ-TREE. DNA data were analyzed using the GTR + G + I model, and amino acid data were analyzed using the LG model. Statistical support for each internal branch of the coalescent-based trees was evaluated using local posterior probability (LPP) values [37].

### 2.4. Concordance Factor

Gene tree support and conflict were evaluated by concordance factor analysis using the IQ-TREE2. Concordance factors were calculated with IQ-TREE2 to quantify the degree of agreement between individual gene trees and the reference (species) topology. Gene concordance factor (gCF) was defined as the percentage of decisive gene trees that contain each internal branch of the reference tree. Site concordance factor (sCF) was defined as the proportion of informative sites that support the focal topology for each internal branch, estimated by quartet sampling around that branch. gCF was computed using command “iqtree2 -t <species tree data> --gcf <gen tree data> --prefix concord”. sCF was computed using command “iqtree2 -t <species tree data> -s <concatenated alignment data> --scf 100,000 --prefix concord”. Both analyses were performed for nucleotide and amino acid data.

### 2.5. Polytomy Test

The polytomy test was performed using the ASTRAL package (option t 10) [4]. This statistical test is based on the multispecies coalescence model, and evaluates the null hypothesis that a branch in the estimated species tree should be replaced by a polytomy.

## 3. Results

### 3.1. Dataset Characteristics

The minimum and maximum alignment lengths for the DNA data were 1098 and 7890 bp (mean: 1763 bp), respectively. The minimum and maximum proportions of variable alignment were 33.5 and 95.2% (mean: 60.1%), respectively. The minimum and maximum alignment lengths for the amino acid data were 334 and 2619 sites (mean: 556 sites), respectively. The minimum and maximum proportions of variable alignment sites were 7.5 and 98.3% (mean: 51.6%), respectively.

The alignment length was 1,530,345 bp, and the number of variable and parsimony-informative sites for the 868 genes in the DNA data were 900,789 (58.9%) and 787,280 (51.4%), respectively. For amino acid sequences, the corresponding values were 510,115 sites, 254,934 variable sites (50%), and 192,410 parsimony-informative sites (37.7%).

### 3.2. Phylogenetic Position of F. commune

**Figure 2:**
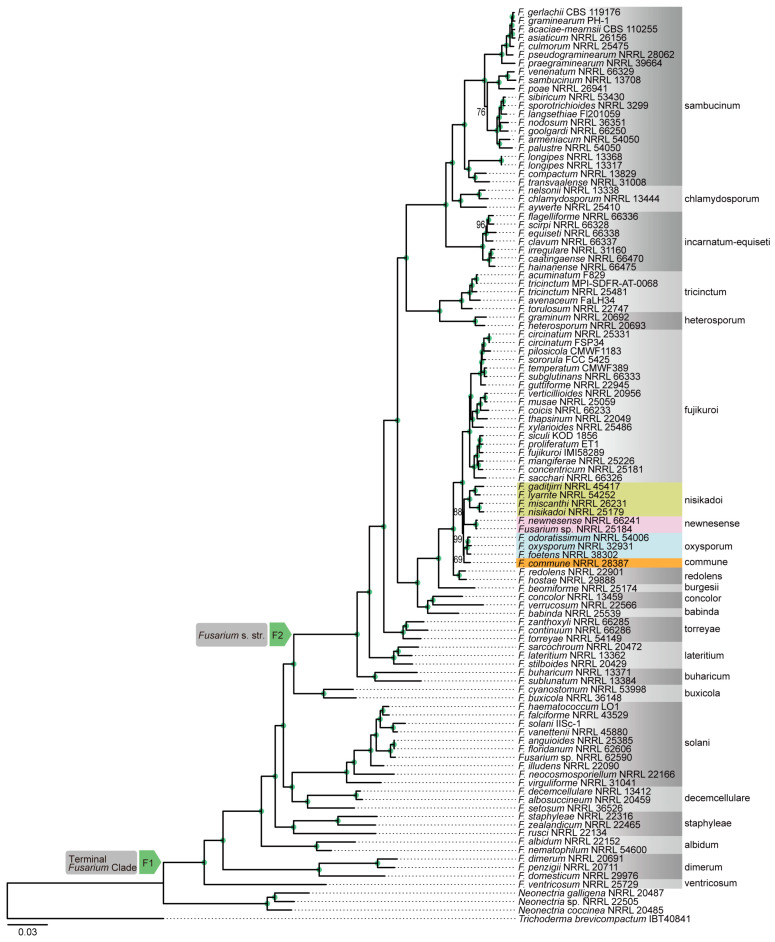
Phylogenetic trees constructed using the concatenation approach in IQ-TREE2 with the amino acid sequences of 868 orthologous genes. Numbers above the internal nodes represent the maximum likelihood of bootstrap (BS) support. Nodes marked with green circles indicate 100 BS value. Node F1 is for the terminal *Fusarium* clade. Node F2 is for the *Fusarium s. str*. clade.

**Figure 3:**
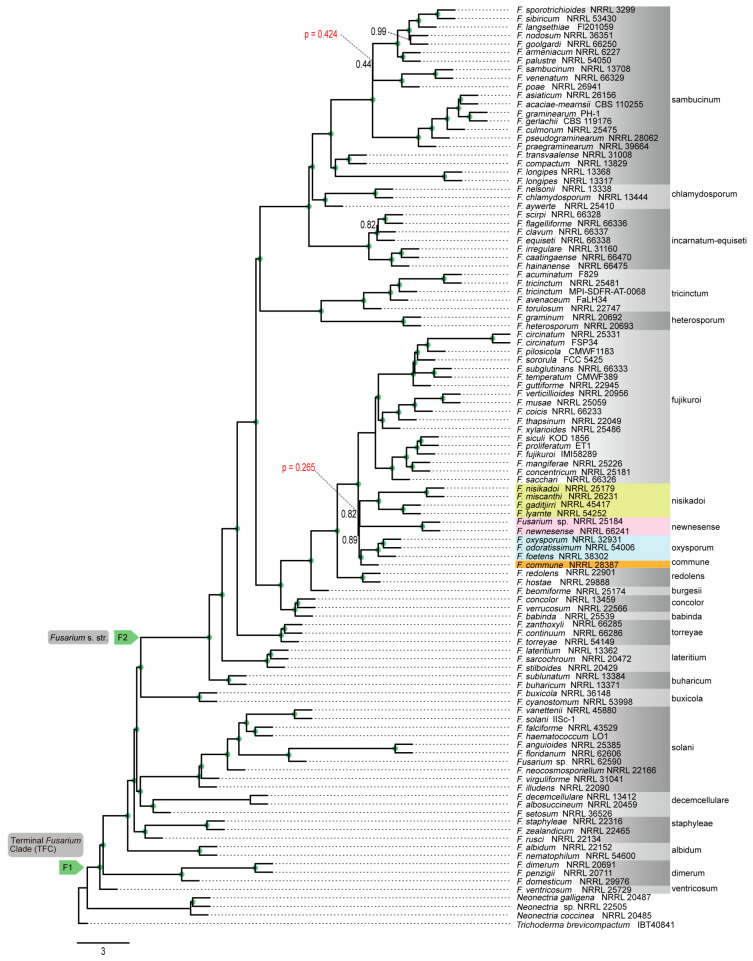
Phylogenetic trees constructed using the coalescent approach in ASTRAL III with the amino acid sequences of 868 orthologous genes. Numbers above the internal nodes represent local posterior probability (LPP) support. Nodes marked with green circles indicate 1 LPP value. Node F1 is for the terminal *Fusarium* clade. Node F2 is for the *Fusarium* s. str. clade.

**Figure 4:**
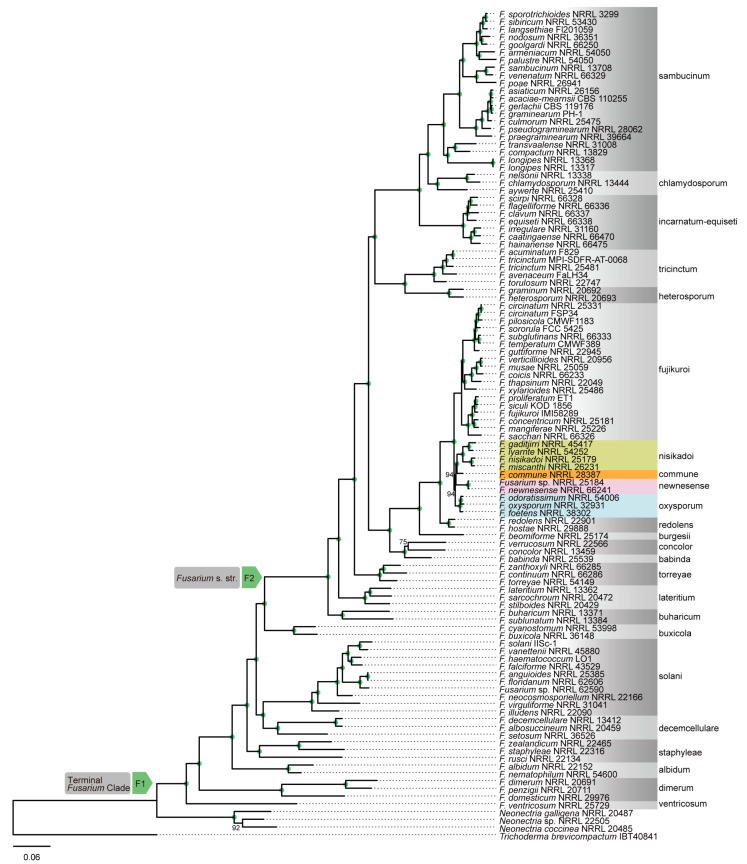
Phylogenetic trees constructed using the concatenation approach in IQ-TREE2 with the DNA sequences of 868 orthologous genes. Numbers above the internal nodes represent the maximum likelihood of bootstrap (BS) support. Nodes marked with green circles indicate 100 BS value. Node F1 is for the terminal *Fusarium* clade. Node F2 is for the *Fusarium s. str.* clade.

**Figure 5:**
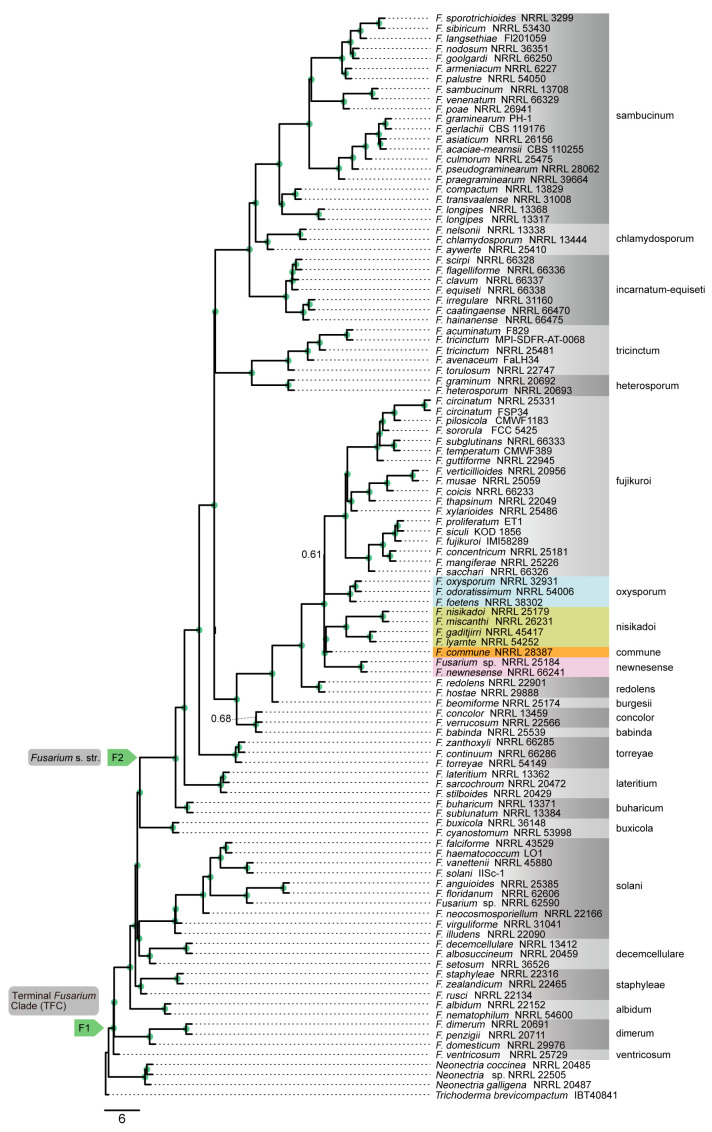
Phylogenetic trees inferred using the coalescent approach in ASTRAL III with the DNA sequences of 868 orthologous genes. Numbers above the internal nodes represent local posterior probability (LPP) support. Nodes marked with green circles indicate 1 LPP value. Node F1 is for the terminal *Fusarium* clade. Node F2 is for the *Fusarium s. str*. clade.

Genome-scale phylogenetic analyses were performed using concatenation and coalescence approaches based on nucleotide and amino acid sequence data from 868 orthologous genes. These analyses revealed that the phylogenetic position of *F. commune* was not consistent between different data type (Figure 2, Figure 3, Figure 4 and Figure 5). In the phylogenetic tree based on the amino acid sequences obtained using concatenation (Figure 2) and coalescent approaches (Figure 3), *F. commune* was sister to the *F. oxysporum* SC (bootstrap value: BS: 100; local posterior probability: LPP: 1). However, in both concatenation and coalescence-based analyses using nucleotide sequences, *F. commune* was sister to *F. nisikadoi* SC (Figure 4 and Figure 5; BS: 100; LPP: 1).

### 3.3. Gene Concordance Factor and Polytomy Test

In analyses based on concatenated amino acid datasets and ASTRAL trees, the split between *F. oxysporum* SC and *F. commune* showed a gene concordance factor (gCF) of 55 and a site concordance factor (sCF) of 14.2 (Supplementary Figures S1 and S2). In contrast, analyses based on concatenated nucleotide datasets and ASTRAL trees yielded gCF and sCF values of 36 and 31, respectively (Supplementary Figures S3 and S4).

The polytomy test confirmed an unreliable branching order among the (*F. nisikadoi* SC and *F. commune*), *F. oxysporum* SC, and *F. newnesense* SC clades in the amino acid-based coalescent tree. Polytomy was detected among these relationships, with a high *p*-value of 0.265 (Figure 4). For the other trees, there was no polytomy at the ancestral node of *F. commune*.

## 4. Discussion

### 4.1. Phylogenetic Reconstruction

Phylogenetic inference using concatenation- and coalescent-based approaches, based on genomic data for various groups of organisms, is now widely used. Concatenation-based phylogenetic analysis is a scaled-up version of traditional multigene analysis, which is automated and utilizes large amounts of data. Therefore, errors, such as paralogous gene sampling and missing data, are typical. However, because these errors occur randomly and infrequently, they tend to be averaged across the dataset, resulting in phylogenetic trees with a high statistical significance.

Nevertheless, recent empirical observations of gene tree discordance, that is, cases in which genes have different evolutionary histories, have shown that the traditional concatenation approach [38] cannot fully account for such discordance among gene trees inferred from phylogenetic data. Consequently, several studies applied coalescent-based methods.

However, because the choice between coalescent- and concatenation-based approaches remains controversial regarding the accuracy of species tree estimation [2,39], both methods were used in the current study. Using both DNA and amino acid data, genome-scale phylogenetic trees were reconstructed to evaluate the monophyly of *Fusarium* SCs and their evolutionary relationships. Comparisons between concatenation- and coalescent-based phylogenetic trees revealed differences in the branching order of certain evolutionary lineages. Likewise, comparisons between DNA- and amino acid-based phylogenetic trees showed topological incongruence (Figure 2, Figure 3, Figure 4 and Figure 5). Both trees had high statistical support for internal branches. As a result, the findings supported the phylogenetic trees proposed by Gómez-Chavarría et al. [10] and Lizcano-Salas et al. [11].

These results demonstrate that, consistent with previous studies, NGS data consistently support the monophyly of SCs across different datasets, except for the placement of *F. commune*.

### 4.2. Phylogenetic Position of F. commune

Since its establishment in 2003 [40], *F. commune* has been reported as a pathogen of multiple agricultural crops, including horseradish, rice, and tomato [41,42,43]. In addition, *F. commune* is recognized as an important pathogen causing severe root rot in conifer seedlings, resulting in substantial economic losses [44].

To date, the phylogenetic placement of *F. commune* has differed even among genome-scale phylogenetic analyses, depending on the data type (nucleotide vs. amino acid sequences) and analytical methods, resulting in two alternative hypotheses placing the species either within the *F. nisikadoi* SC or the *F. oxysporum* SC (Figure 1) [10,11,12]. Our results are consistent with previous studies in demonstrating that the phylogenetic position of *F. commune* varies depending on the type of sequence data analyzed.

In phylogenetic trees inferred from amino acid sequence data, *F. commune* was a sister lineage to the *F. nisikadoi* SC (Figure 2 and Figure 3), whereas in trees based on nucleotide sequence data, it was regarded as sister to the *F. oxysporum* SC (Figure 4 and Figure 5). Concordance factor analyses further clarified the nature of this discordance (Supplementary Figures S1–S3). In analyses based on concatenated amino acid datasets and ASTRAL trees, the split between *F. oxysporum* SC and *F. commune* showed a gene concordance factor (gCF) of 55 and a site concordance factor (sCF) of 14.2. This result indicates that more than half of the genes support this bifurcation, suggesting a degree of consistency at the gene level. However, the lack of complete agreement among genes implies substantial gene tree discordance at this node. Notably, the sCF value was far below the random expectation (33%), indicating that individual alignment sites do not support this branching pattern. This finding suggests that the split represents an ancient divergence associated with a short internal branch, resulting in limited phylogenetic signal at the site level, or that incomplete lineage sorting (ILS) has diluted the phylogenetic signal.

In contrast, analyses based on concatenated nucleotide datasets and ASTRAL trees yielded gCF and sCF values of 36 and 31, respectively, for the split between *F. nisikadoi* SC and *F. commune*. In this case, fewer than half of the genes supported the bifurcation, indicating weak support at the gene level. Furthermore, the sCF value was close to the random expectation (sCF = 33), suggesting that nucleotide sites do not preferentially support a specific phylogenetic relationship at this node. Collectively, these results demonstrate that the phylogenetic placement of *F. commune* is highly sensitive to data type, and that it does not represent a lineage fixed by a single, well-supported bifurcation. In particular, its relationships with *F. nisikadoi* SC and *F. oxysporum* SC appear to be strongly influenced by gene tree discordance associated with rapid diversification processes. In addition, the polytomy test based on amino acid sequence data indicated that the topology ((*F. commune*, *F. oxysporum* SC), *F. newnesense* SC, *F. nisikadoi* SC) represents a polytomous relationship, suggesting an evolutionarily unresolved lineage that diverged over a short time span. This pattern implies a complex evolutionary history shaped by incomplete lineage sorting (ILS) and/or gene flow among closely related lineages, and is consistent with the results of the concordance factor analyses. Considering both our results and previous studies—where *F. commune* was alternatively assigned to *F. nisikadoi* SC or *F. oxysporum* SC but consistently positioned in ancestral lineages rather than nested within either SC [10,11]—*F. commune* is treated as an independent lineage, distinct from the currently recognized SCs.

Species identification within the genus *Fusarium* is typically conducted at the level of SCs. Therefore, demonstrating that *F. commune* represents a lineage distinct from other SCs is a significant finding from both taxonomic and diagnostic perspectives. As noted above, *F. commune* is an agriculturally important plant pathogen, and its accurate classification is essential for the diagnosis of diseases and the development of effective management strategies. Moreover, this finding not only contributes to the accurate identification and control of diseases caused by *F. commune*, but also enhances our understanding of the evolutionary diversity within the genus *Fusarium*.

## 5. Conclusions

High-throughput sequencing has enabled the use of large gene sequence datasets for extensive taxon sampling. The phylogenetic position of Fusarium commune was found to differ depending on the data type: it appeared as a basal lineage of the *F. oxysporum* SC in amino acid-based phylogenies, whereas it was placed as a basal lineage of the *F. nisikadoi* SC in nucleotide-based phylogenies. This phylogenetic instability was further supported by concordance factor analysis, and a polytomy was detected at the ancestral node of F. commune in the coalescent-based tree inferred from amino acid sequences. Based on these findings, we consider *F. commune* to be evolutionarily independent of the other SCs. This study provides a common framework for the phylogenetic treatment of *F. commune* at the SC level and contributes to applied research such as species identification.

## Data Availability

The genomic data used in this study are available in the NCBI database with reference to the GenBank accession numbers in Supplementary Table S1.

## Supplementary Materials

The following supporting information can be downloaded at: https://www.mdpi.com/article/10.3390/jof12020112/s1, Table S1: GenBank accessions for genome sequence; Figure S1: Cladogram based on amino acid data of 868 genes using concatenation-approach analyzed with ASTRAL with concordance factors; Figure S2: Cladogram based on amino acid data of 868 genes using coalescent-approach analyzed with ASTRAL with concordance factors; Figure S3: Cladogram based on amino acid data of 868 genes using concatenation-approach analyzed with ASTRAL with concordance factors; Figure S4: Cladogram based on amino acid data of 868 genes using coalescent-approach analyzed with ASTRAL with concordance factors.

## Abbreviations

The following abbreviations are used in this manuscript:

SC	species complex
LPP	local posterior probability
BS	bootstrap
