# Supplementary material for: Genome-Scale Phylogenetic Analyses Provide Insights into the Phylogenetic Placement of Fusarium commune
Source: J Fungi (Basel). 2026 Feb 5;12(2):112. doi: 10.3390/jof12020112 (PMC12942637; doi:10.3390/jof12020112)
Supplement: Supplementary file 1 [file jof-12-00112-s001.zip › Supplementary Figure S4_AstralTreeNucl_phypart.pdf]

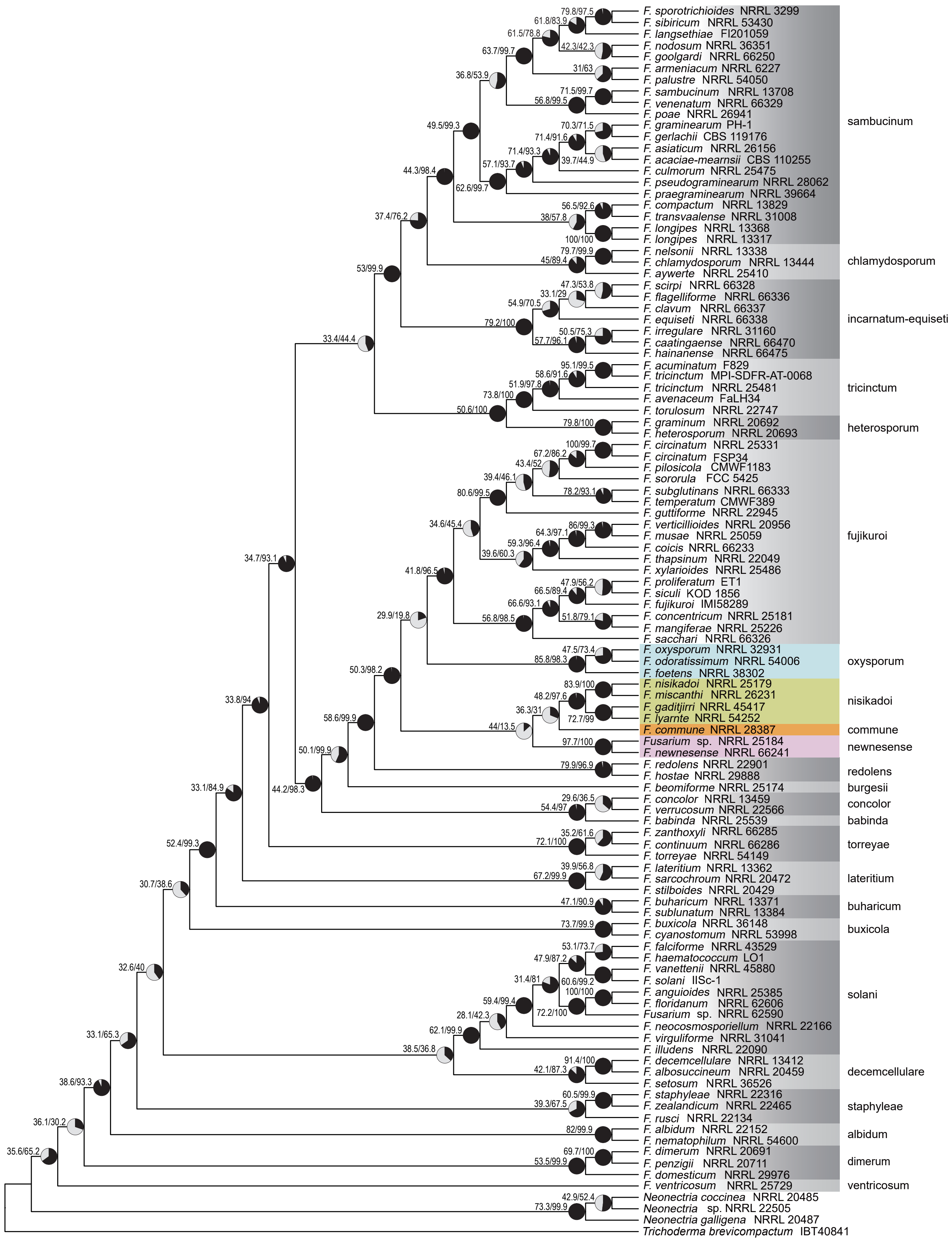

Supplementary Figure S4 Cladogram based on amino acid data of 868 genes using coalescent-approach analyzed with ASTRAL with con. The number on nodes indicates sCF/gCF. The pie charts at each node present the gCF.
