# Supplementary material for: Genome-Scale Phylogenetic Analyses Provide Insights into the Phylogenetic Placement of Fusarium commune
Source: J Fungi (Basel). 2026 Feb 5;12(2):112. doi: 10.3390/jof12020112 (PMC12942637; doi:10.3390/jof12020112)
Supplement: Supplementary file 1 [file jof-12-00112-s001.zip › Supplementary Table S1.pdf]

Supplemental Table S1 GenBank accessions for genome sequences

Species	Strain no.	Assembly accession	Status of strain	Reference
Fusarium acaciae-mearnsii	CBS 110255	GCA_017657105		[6]
F. acuminatum	F829	GCA_013363215		[13]
F. albidum	NRRL 22152	GCA_013618265	Type	[14]
F. albosuccineum	NRRL 20459	GCA_012931995		[14]
F. anguioides	NRRL 25385	GCA_012977745	Neotype	[14]
F. armeniacum	NRRL 6227	GCA_013623825		[14]
F. asiaticum	NRRL 26156	GCA_013618305	Type	[14]
F. avenaceum	FaLH27	GCA_000769295		[14]
F. aywerte	NRRL 25410	GCA_013186375		[14]
F. babinda	NRRL 25539	GCA_013184435		[14]
F. beomiforme	NRRL 25174	GCA_002980475		[14]
F. buharicum	NRRL 13371	GCA_014822075		[15]
F. buxicola	NRRL 36148	GCA_014899095		[14]
F. caatingaense	NRRL 66470	GCA_013624355		[16]
F. chlamydosporum	NRRL 13444	GCA_014898915		[14]
F. circinatum	NRRL 25331	GCA_013396185	Type	[14]
F. circinatum	FSP 34	GCA_000497325		[17]
F. clavum	NRRL 66337	GCA_004367155		[18]
F. coicis	NRRL 66233	GCA_013781345	Type	[19]
F. commune	NRRL 28387	GCA_013618355		[14]
F. compactum	NRRL 13829	GCA_013618385		[14]
F. concentricum	NRRL 25181	GCA_014824425	Type	[20]
F. concolor	NRRL 13459	GCA_013184415	Synonym type	[14]
F. continuum	NRRL 66286	GCA_013184455	Type	[14]
F. culmorum	NRRL 25475	GCA_013618375	Type	[14]
F. cyanostomum	NRRL 53998	GCA_014824385		[14]
F. decemcellulare	NRRL 13412	GCA_013266205		[14]
F. dimerum	NRRL 20691	GCA_013623525		[14]
F. domesticum	NRRL 29976	GCA_013618395		[14]
F. equiseti	NRRL 66338	GCA_004367125		[14]
F. falciforme	NRRL 43529	GCA_013363125		[14]
F. flagelliforme	NRRL 66336	GCA_004367175		[18]
F. floridanum	NRRL 62606	GCA_003947005		[21]
F. foetens	NRRL 38302	GCA_013623845		[14]
F. fujikuroi	IMI58289	GCA_900079805		[14]
F. gaditjirrii	NRRL 45417	GCA_013266175		[14]
F. gerlachii	CBS 119176	GCA_017656835		[22]

Supplemental Table S1 Cont.

Species	Strain no.	Assembly accession	Status of strain	Reference
F. guttiforme	NRRL 22945	GCA_013186795		[23]
F. goolgardi	NRRL 66250	GCA_014899075	Type	[19]
F. graminearum	PH-1	GCA_000240135		[14]
F. graminum	NRRL 20692	GCA_013266165		[14]
F. haematococcus	LO1	GCA_010015875		[24]
F. hainanense	NRRL 66475	GCA_013618405		[14]
F. heterosporum	NRRL 20693	GCA_013396295		[14]
F. hostae	NRRL 29888	GCA_013184365	Type	[14]
F. illudens	NRRL 22090	GCA_013623515		[14]
F. irregulare	NRRL 31160	GCA_004367085		[14]
F. langsethiae	Fl201059	GCA_001292635		[14]
F. lateritium	NRRL 13362	GCA_014898835		[14]
F. longipes	NRRL 13368	GCA_013618485		[14]
F. longipes	NRRL 13317	GCA_013618495		[14]
F. lyarnte	NRRL 54252	GCA_014898885		[14]
F. mangiferae	NRRL 25226	GCA_013758935		[14]
F. miscanthi	NRRL 26231	GCA_014898875		[14]
F. musae	NRRL 25059	GCA_013623345		[25]
F. nelsonii	NRRL 13338	GCA_014898925		[14]
F. nematophilum	NRRL 54600	GCA_013623595	Type	[14]
F. neocosmosporiellum	NRRL 22166	GCA_006518225	synonym type	[14]
F. newnesense	NRRL 66241	GCA_013184375	Type	[14]
F. nisikadoi	NRRL 25179	GCA_013623555		[14]
F. nodosum	NRRL 36351	GCA_014898975		[26]
F. odoratissimum	NRRL 54006	GCA_000260195		[13]
F. oxysporum	NRRL 32931	GCA_000271745		[14]
F. palustre	NRRL 54050	GCA_014899045		[27]
F. penzigii	NRRL 20711	GCA_013623535		[14]
F. pilosicola	CMWF1183	GCA_020615335	Type	[28]
F. poae	NRRL 26941	GCA_013623615	Type	[14]
F. praegraminearum	NRRL 39664	GCA_002093855	Type	[14]
F. proliferatum	ET1	GCA_900067095		[29]
F. pseudograminearum	NRRL 28062	CGA_000303195		[14]
F. redolens	NRRL 22901	GCA_014899085		[14]
F. rusci	NRRL 22134	GCA_017140155		[14]
F. sacchari	NRRL 66326	GCA_013759005		[14]
F. sambucinum	NRRL 13708	GCA_014899025		[14]

Supplemental Table S1 Cont.

Species	Strain no.	Assembly accession	Status of strain	Reference
F. sarcochroum	NRRL 20472	GCA_013266185		[14]
F. scirpi	NRRL 66328	GCA_004367495		[14]
F. setosum	NRRL 36526	GCA_013623625		[14]
F. sibiricum	NRRL 53430	GCA_014898995		[28]
F. siculi	KOD 1856	GCA_019843635		[6]
F. solani	IISc-1	GCA_013168735		[30]
F. sororula	FCC 5425	GCA_017579625		[27]
Fusarium sp.	NRRL 25184	GCA_013755755		[14]
	NRRL 62590	GCA_003947015		[14]
F. sporotrichiodes	NRRL 3299	GCA_003012315		[14]
F. staphyleae	NRRL 22316	GCA_017140175		[14]
F. stilboides	NRRL 20429	GCA_014822085		[14]
F. subglutians	NRRL 66333	GCA_013396075		[14]
F. sublunatum	NRRL 13384	GCA_013623665		[14]
F. temperatum	CMWF389	GCA_001513835		[14]
F. thapsinum	NRRL 22049	GCA_013186935		[14]
F. torreyae	NRRL 54149	GCA_014824505		[14]
F. torulosum	NRRL 22747	GCA_013623875		[14]
F. transvaalense	NRRL 31008	GCA_013623685		[14]
F. tricinctum	MPI-SDFR-AT-0068	GCA_020744515		[24]
F. tricinctum	NRRL 25481	GCA_012977725	Type	[14]
F. vanettenii	NRRL 45880	GCA_000151355		[14]
F. venenatum	NRRL 66329	GCA_013623635		[14]
F. ventricosum	NRRL 25729	GCA_013623725	Type	[14]
F. verrucosum	NRRL 22566	GCA_013623715		[14]
F. verticillioides	NRRL 20956	GCA_900007375		[14]
F. virguliforme	NRRL 31041	GCA_013363175		[14]
F. xylarioides	NRRL 25486	GCA_013623735	Type	[14]
F. zanthoxyli	NRRL 66285	GCA_013623745	Type	[14]
F. zealandicum	NRRL 22465	GCA_013266195		[14]
Neonectria coccinea	NRRL 20485	GCA_013757005		[14]
Neonectria galligena	NRRL 20487	GCA_013759035		[14]
Neonectria sp.	NRRL 22505	GCA_013756995		[14]
Trichoderma brevicompactum	IBT40841	GCA_003012085		[14]
